# Amalgam Restorations

**Published:** 1919

**Authors:** 


***Amalgam Restorations.*** By Lieut.-Col. NEIL J. McCOLLUM. *Journal Association Military Dental Surgeons of U. S.*, October, 1918.

The author recommends the use of amalgam fillings in the Army as being the means of saving many teeth. In preparing cavities Black's eight rules of cavity preparation are carried out wherever practicable. The cavity is dried and kept dry during insertion of the filling. The alloy is thoroughly amalgamated with as little excess mercury as possible. Filling is packed with flat-faced instruments; no burnisher used. Fillings are carved to conform to the natural outlines of the tooth. Fillings are polished at a subsequent sitting, if possible, or after allowing time for crystallization at the same sitting.

